# Altered ocular microvasculature in patients with systemic sclerosis and very early disease of systemic sclerosis using optical coherence tomography angiography

**DOI:** 10.1038/s41598-022-14377-6

**Published:** 2022-06-29

**Authors:** Nataša Mihailovic, Larissa Lahme, Sonja Braasch, Friederike Rosenberger, Nicole Eter, Jan Ehrchen, Maged Alnawaiseh

**Affiliations:** 1 Department of Ophthalmology, University of Muenster Medical Center, Domagkstraße 15, Muenster, Germany; 2 Department of Ophthalmology, Klinikum Fulda, University of Marburg, Campus Fulda, Fulda, Germany; 3 Department of Dermatology, University of Muenster, Muenster, Germany

**Keywords:** Rheumatology, Eye diseases

## Abstract

The vascular hypothesis of systemic sclerosis (SSc) would predict microvascular alterations should also affect anatomical regions like ocular microvasculature. The objective of this study was to evaluate retinal and choriocapillary vessel density (VD) in patients with definite SSc and very early disease of systemic sclerosis (VEDOSS) using optical coherence tomography angiography (OCTA). 22 eyes of 22 patients and 22 eyes of 22 healthy subjects were included in this study. Patients were classified into patients with definite SSc and patients with VEDOSS. VD data of the superficial OCT angiogram (OCTA-SCP), deep OCT angiogram (OCTA-DCP) and choriocapillaris (OCTA-CC) were analysed. VD in the OCTA-SCP and OCTA-CC was lower in patients with SSc (p < 0.05). In VEDOSS patients, VD in the OCTA-CC was still reduced compared to controls (p < 0.05). Correlation analysis revealed a positive correlation between nailfold capillaroscopy and VD of OCTA-CC (Spearman correlation coefficient (rSp) 0.456, p < 0.05) and a negative correlation between skin score and VD of OCTA-SCP (p < 0.05). Ocular perfusion seems to be impaired in patients with SSc and even VEDOSS. VD correlated with disease severity. OCTA could be a new useful diagnostic and predictive parameter for monitoring patients with different stages of the disease.

## Introduction

Systemic sclerosis (SSc) is a rare multisystem disease characterized by microvascular damage, activation of innate and adaptive immunity, production of autoantibodies and sclerosis of the skin and many other organs. Diffuse cutaneous SSc (dcSSc) and limited cutaneous SSc (lcSSc) present the two major subsets of SSc. Despite encouraging developments in recent years, the therapy of SSc is still difficult, especially in patients in the later stages of disease which often suffer from extensive fibrosis^1^.

Vasculopathy is an important early step in the pathogenesis of SSc: most patients initially experience Raynaud’s phenomenon (RP), a painful, cold-induced vasospasm of small arteries usually localized in fingers and toes, as first disease symptom^2^. It has been demonstrated that structural changes exist in the microvasculature before inflammation and fibrosis are clinically evident in patients who develop SSc. Indeed, these alterations can independently predict development of SSc in patients with RP^3^. Based on these findings the concept of a very early diagnosis of systemic sclerosis (VEDOSS) was developed implying patients with RP and detection of disease specific autoantibodies or pathologic changes in nailfold capillaroscopy but no disease symptoms other than puffy fingers or arthritis^4,5^. A recently published large single cohort study by Siquiera et al. found the combinations most associated with progression to SSc were RP, puffy fingers, positive antinuclear antibodies (ANA) and a scleroderma pattern at nailfold capillaroscopy (SD-NFC) and/or SSc specific antibody^6^.

The vascular hypothesis of SSc pathophysiology, however, would predict that changes in the microcirculation are not only evident in the nailfolds but are rather a generalized phenomenon which should affect other anatomical regions including the oral and ocular microvasculature and especially the choroid, as one of the most highly vascularized tissues of the human body^7^. Ocular symptoms in patients with SSc are diverse and reach from keratoconjunctivitis sicca and blepharitis, eyelid abnormalities, pterygium, orbital and conjunctival varices, cataract formation, cornea guttata and glaucoma to retinal diseases like central vein occlusion or drusen^8^.

Until the beginning of the last decade, studies on the chorioretinal microvasculature could only be performed invasively by fluorescein or indocyanine green angiography. With optical coherence tomography angiography (OCTA), an innovative technology, chorioretinal vasculature can now be visualized in a non-invasive and dyeless way. Particularly, a quantification of the chorioretinal perfusion including the perfusion of the optic nerve head is possible^9^. In their pilot study, Rothe et al. demonstrated a reduced perfusion of the superficial capillary plexus (SCP) in patients with SSc compared to healthy controls using OCTA. In the following, their research group also demonstrated a reduction of the submacular perfusion of the choriocapillaris^10,11^. However, there are still limited data on choroidal, retinal and optic nerve head microvascular perfusion in patients with SSc and we are not aware of any OCTA data on retinal and choriocapillary microvasculature in patients with VEDOSS^12^.

Thus, we conducted this study analyzing retinal and choriocapillary perfusion measured using OCTA in patients with SSc and VEDOSS.

## Materials and methods

22 eyes of 22 patients with SSc, according to the 2013 ACR/EULAR criteria or the VEDOSS criteria and 22 eyes of 22 gender and age matched healthy controls were consecutively included in this study^13^. For subgroup analysis, the SSc group was further divided into patients with either a dcSSc or lcSSc (definite SSc, n = 15) and patients with VEDOSS (n = 7).

The study was approved by the Ethics Committee of the University of Muenster, North Rhine Westphalia, Germany. Before performing any ophthalmic examination, the study protocol was explained in detail and all participants signed an informed consent form. The study adhered to the tenets of the Declaration of Helsinki.

Patients and controls with high myopia or media opacities preventing high-quality imaging like cataract and cornea guttata, vitreoretinal disease, previous retinal surgery, macular edema, glaucoma or neurological disease were excluded from the study.

All study participants underwent an ophthalmic examination including refraction, best-corrected visual acuity, anterior segment examination, binocular fundus examination and OCTA imaging. None of the patients showed clinical signs of intraocular or retinal involvement. Ocular surface and eyelid abnormalities were evaluated for each patient. As a well-validated surrogate measure of disease severity skin thickness was determined using the modified Rodnan skin score (mRSS), which was determined by an expert examiner (JE and SB). It entails a 17-point assessment of skin thickness on various areas of the body, culminating in a 51-point maximum scale of severity^14^. The mRSS is currently considered the most appropriate technique and is a validated primary outcome measure in clinical trials of patients with dcSSc and lcSSc.

Nailfold capillaroscopy was performed by the same expert examiners in patients and controls (JE and SB) in 3 fingers of both hands. Characteristics for a scleroderma pattern in nailfold capillaroscopy [capillary dimensions (especially presence of giant capillaries), abnormal capillary shapes and haemorrhages^15^] were evaluated semi-quantitatively. For quantitative analysis and for comparison to the OCTA data we calculated the mean capillary density per millimetre.

### Optical coherence tomography angiography (OCTA)

OCTA imaging was performed using the RTVue XR Avanti device (Optovue Inc, Fremont, California, USA). Integrated split-spectrum amplitude-decorrelation angiography (SSADA) was used to generate the angiographic data. OCTA imaging technology has been described in detail elsewhere^16^. The macula was imaged using a 3 × 3 mm OCTA scan, the optic nerve head OCT-angiogram was obtained using the 4.5 × 4.5 mm scan. All examinations were performed under the same conditions at the same location by an expert examiner.

The automated segmentation was checked for proper segmentation by an experienced reader (NM, MA) before data analysis. In cases of incorrect segmentation, the image was excluded. Images with poor signal strength (signal strength index < 6) or with motion artifacts preventing reliable image analysis were not included in the study, either. The en face OCT angiogram of the superficial capillary plexus (OCTA-SCP) was segmented with an inner boundary 3 μm below the internal limiting membrane (ILM) and an outer boundary set at 15 μm below the inner plexiform layer (IPL). The deep capillary plexus (DCP) en face OCT-A image was segmented with an inner boundary 15 μm below the IPL and an outer boundary at 70 μm below the IPL. Choriocapillaris (CC) segmentation was defined from 10 μm above to 30 μm below Bruch’s membrane.

After imaging, the VD of the OCTA-SCP, OCTA-DCP, the radial peripapillary capillary density (RPC) and the foveal avascular zone (FAZ) area were analyzed using the integrated device software. To determine VD values of the OCT angiogram of the choriocapillaris (OCTA-CC), OCTA images were exported. Using Adobe Photoshop CS6 (Adobe Systems, Inc., California) images were converted into grey scales. Each pixel was attributed to a value that represents the strength of the decorrelation signal. VD of the choriocapillaris was calculated as the mean decorrelation value of all pixels in the images^17^.

### Statistics

IBM SPSS^®^ Statistics 26 for Windows (IBM Corporation, Somers, NY, USA) and GraphPad Prism 5 (GraphPad Software Inc., San Diego, CA, USA) were used for statistical analyses. The data was tested for normality distribution using the Shapiro–Wilk test and did not fit a normal distribution. The descriptive data are therefore presented as median [25th; 75th percentile]. The two groups were compared using the Mann–Whitney U test for non-normally distributed variables, and the degree of correlation between two variables was expressed as the Spearman’s correlation coefficient (rSp). To exclude a significant influence of arterial hypertension on the OCTA parameters, a linear regression analysis was performed. Inferential statistics are intended to be exploratory (hypothesis-generating), not confirmatory, and are interpreted accordingly. The comparison-wise type-I error rate is controlled instead of the experiment-wise error rate. The chosen level of significance was p < 0.05.

### Ethical approval

All participants in the study gave their written informed consent according to Declaration of Helsinki. The study was approved by the Ethics Committee of the University of Muenster, North Rhine Westphalia, Germany (2015-402-f-S).

## Results

There was no statistically significant difference in age between the study and control group (p = 0.42). The demographic characteristics of patients and healthy controls are shown in Table 1. Detailed clinical and laboratory data as well as ocular symptoms of each patient are shown in the supplementary material (Supplementary Table 1). 8 out of 22 patients and 2 out of 22 healthy controls suffered from arterial hypertension (aHT) which is known to affect OCTA metrics^18^. Linear regression analysis revealed no statistically significant influence of aHT on the presented OCTA data except for the parameter *SCP parafovea* (p < 0.05)*.*

**Table 1:** Age and gender distribution of the systemic sclerosis (SSc) group and control group. Data are presented as median [25th, 75th percentile].

	SSc group	Control group
n(^a^) dcSSc/lcSSc	22 (7) 8/7	22 Not applicable
Age (years)	54.59 [46.75, 61.25]	51.59 [44.75, 49.50]
Gender (f/m)	16/6	16/6

^a^Subgroup of patients with very early disease (VEDOSS); dcSSc = patients with diffuse cutaneous SSc; lcSSc = patients with limited cutaneous SSc.

**Figure 1:**
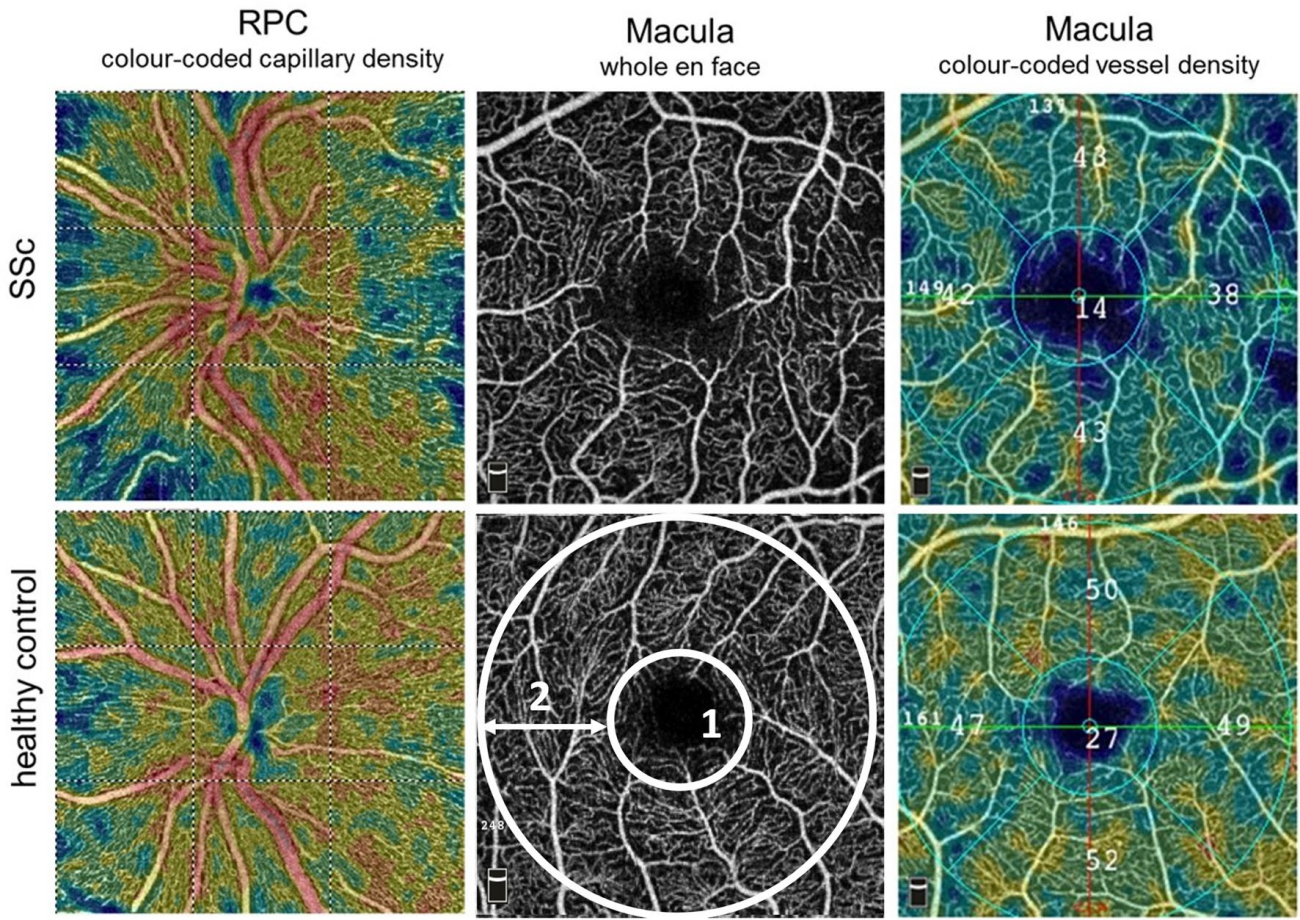
Optical coherence tomography angiography (OCTA). En face OCTA images of the macula (superficial 3 × 3 mm OCT angiogram) and optic nerve head (RPC = radial peripapillary capillary 4.5 × 4.5 mm) of a patient with definite systemic sclerosis (SSc, top row) and an age-matched healthy control (bottom row). The foveal vessel density (1) (diameter of 1 mm, small white circle), parafoveal vessel density (2) (diameter from 1 to 2.5 mm, big white circle) and *whole en face* vessel density [the average vessel density of the entire 2.5 mm circle (1 and 2)] were analyzed.

The VD in the OCTA-SCP (*whole en face*), the OCTA-CC and the RPC (*inside disc*) of the SSc group (n = 22) was significantly lower compared to the healthy control group (OCTA-SCP: SSc group: 43.10% [39.95%, 46.90%], control group: 45.25% [43.90%, 48.23%], p = 0.036; OCTA-CC: SSc group: 111.07% [104.39%, 114.61%], control group: 116.96% [114.19%, 118.74%], p = 0.001; RPC: SSc group: 47.05% [42.08%, 53.35%], control group: 52.80% [49.68%, 55.05%], p = 0.021; Fig. 1).

The VD in the OCTA-DCP and FAZ did not differ between both groups (p > 0.05). The VD and FAZ data are summarized in Table 2.

**Table 2:** Vessel density (VD, %) of the SSc group and control group obtained in the regions indicated [superficial retinal capillary plexus (SCP), deep retinal capillary plexus (DCP), choriocapillaris (CC) and optic nerve head capillary density (RPC)], as well as the foveal avascular zone area (FAZ) in mm^2^ and nailfold capillary density (NCD) per millimeter. Data are presented as median [25th, 75th percentile]. A Mann–Whitney-U-test was used to compare both groups. Bold: statistically significant differences.

	OCTA	SSc group	Control group	p-value
SCP (VD)	Whole en face	43.10 [39.95, 46.90]	45.25 [43.90, 48.23]	**0.036**
	Fovea	17.25 [11.40, 18.73]	18.55 [15.58, 22.08]	0.078
	Parafovea	46.10 [42.15, 49.65]	47.60 [46.15, 50.25]	0.089
DCP (VD)	Whole en face	49.50 [45.87, 51.88]	49.40 [47.27, 52.60]	0.573
	Fovea	32.85 [27.32, 36.83]	35.80 [28.10, 40.28]	0.366
	Parafovea	50.55 [48.30, 54.13]	51.15 [49.10, 55.20]	0.589
RPC (VD)	Whole en face	49.90 [47.63, 51,55]	50.75 [48.48, 51.55]	0.526
	Inside disc	47.05 [42.08, 53.35]	52.80 [49.68, 55.05]	**0.021**
	Peripapillary	53.10 [50.75, 54.70]	53.00 [50.88, 54.60]	0.991
CC (VD)	Whole en face	111.07 [104.39, 114.61]	116.96 [114.19, 118.74]	**0.001**
FAZ		0.26 [0.18, 0.33]	0.24 [0.17, 0.31]	0.474
NCD		7.64 [7.0; 8.25]	4.0 [3.125; 5.0]	** < 0.001**

**Figure 2:**
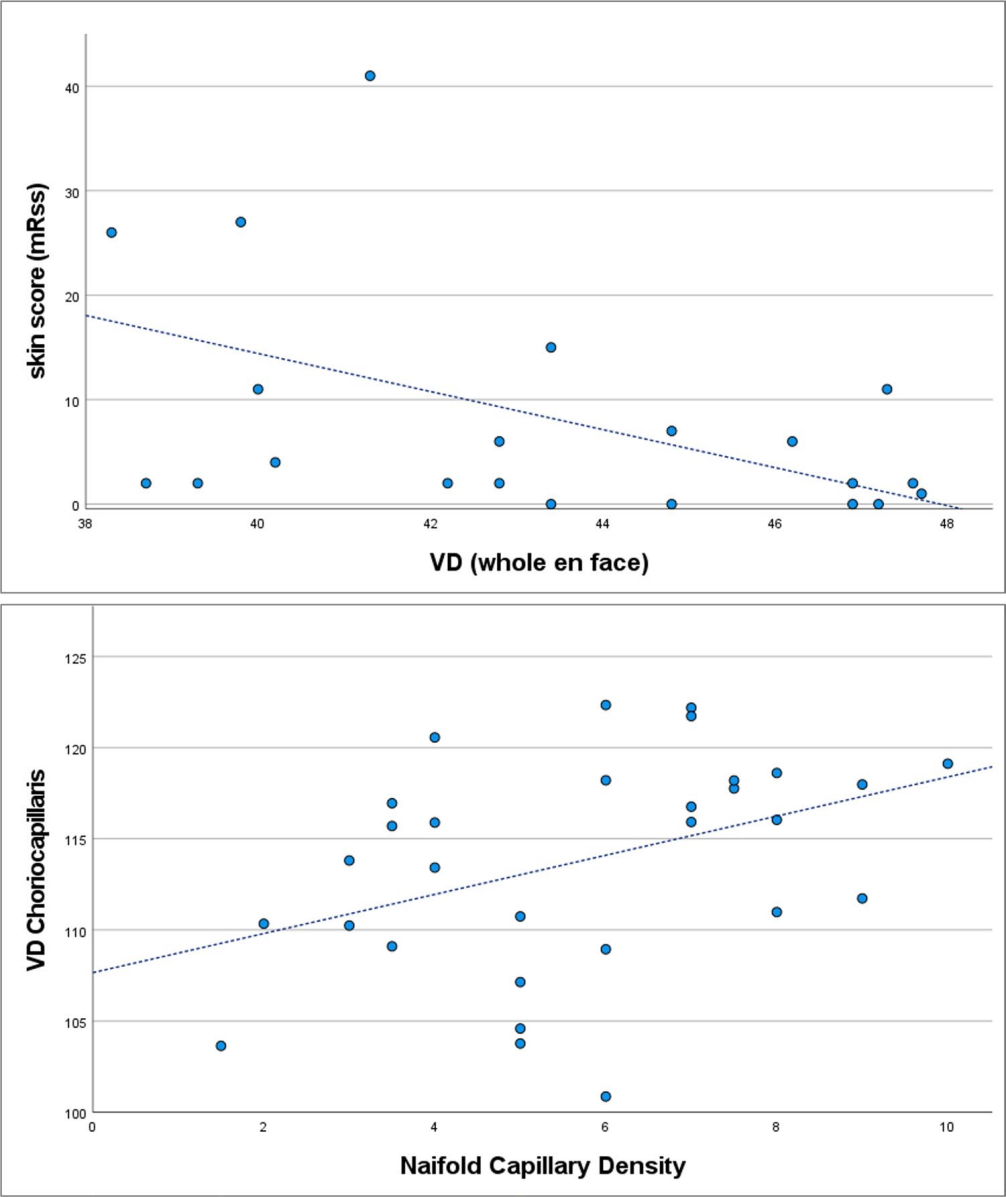
Correlation analysis. Correlation analysis between (**A**) the vessel density (VD) obtained in the superficial OCT angiogram of the macula (whole en face) and the modified Rodnan skin score (mRSS) (Spearman coefficient (rSp) = − 0.504; p = 0.017), and (**B**) the VD of the choriocapillaris and the nailfold capillary density (rSp = 0.456, p = 0.011).

Nailfold capillaroscopy data revealed a significant difference in capillary density between both groups (controls: 7.64/mm [7.0; 8.25]; patients: 4.0/mm [3.125; 5.0]; p < 0.001, Table 2). Correlation analysis showed a statistically significant correlation between the capillary density by nailfold capillaroscopy and the VD of the choriocapillaris (rSp = 0.456, p = 0.011) and a significant negative correlation between the mRSS and the VD obtained in the OCTA-SCP (*whole en face*) (rSp = − 0.504; p = 0.017) (Fig. 2). The correlation between the capillary density by nailfold capillaroscopy and VD of the OCTA-SCP (*whole en face*) was not significant (rSp = 0.342, p = 0.064, Supplementary Table 2).

When comparing patients with VEDOSS (n = 7) and healthy controls, the VD in the OCTA-CC was still significantly reduced (VEDOSS: 107.14% [103.77%, 113.42%], healthy controls: 116.96% [114.19%, 118.74%], p = 0.008). A comparison of the VD data between patients with VEDOSS and patients with definite SSc (n = 15) revealed a significantly reduced VD of the RPC in patients with definite SSc (RPC *whole en face*: SSc: 49.40% [47.30%, 51.00%], VEDOSS: 51.10% [50.50%, 53.00%] p = 0.017). The whole subgroup analysis is shown in the supplemental material (Supplementary Table 3).

## Discussion

Our results suggest for the first time an involvement of the ocular microvasculature even in patients with early stages of SSc (VEDOSS). In patients with definite SSc, we demonstrated alterations of the microvasculature in the SCP, optic nerve head and choriocapillaris. In patients with VEDOSS, we found a reduced VD of the choriocapillaris. Our results are in line with previous OCTA studies in the literature^11,19,20^. Rothe et al. also demonstrated a reduced macular VD in SSc patients with the changes being mainly located in the SCP^11^. In contrast to chat, Carnevali et al. showed an impairment of the microvasculature in SSc patients mainly in the DCP, the VD in the SCP was only reduced in patients with SSc and digital ulcers^19^. However, these studies did not differentiate between patients with SSc and VEDOSS^10^.

The vascular hypothesis of SSc postulates that vasculopathy is the initial pathophysiological event which triggers inflammation and subsequent fibrosis during SSc disease progression^21,22^. Microvascular alterations are usually assessed by studying nailfold capillaries^15^. In patients with RP, the presence of typical changes in nailfold capillaroscopy, especially capillary enlargement and capillary density, were shown to correlate with the risk of developing systemic disease^3^. Our results, too, show a significant correlation between capillary density obtained by nailfold capillaroscopy and VD of the choriocapillaris. The early identification of these patients at risk for the development of SSc is important for targeting therapies to those patients which have the best chance to respond to treatment (“window of opportunity”)^23^. Predictors of progression to SSc in patients with VEDOSS were evaluated in a study by Siquiera et al. and the strongest predictors of progression were combinations of VEDOSS characteristics like RF, puffy fingers, positive ANA and scleroderma pattern in nailfold capillaroscopy^6^. Therefore, it is advised to perform nailfold capillaroscopy in all patients with RP in order to identify VEDOSS. However, a thorough training of the investigator is mandatory to reliably perform nailfold capillaroscopy, which might hamper reproducibility and prognostic value of this technique in clinical practice^24^. In contrast to that, the interobserver reproducibility, repeatability and agreement of OCTA images are very high^25,26^. Moreover, OCTA can be performed fast and easily in clinical practice. Many studies in the literature analyzed the utility of OCTA in assessing the microcirculation in different systemic diseases^27–30^.

In this study we found a significantly reduced VD of the choriocapillaris already in VEDOSS patients. Thus, it is tempting to speculate that OCTA imaging—similarly to nailfold capillaroscopy—could also be used to evaluate the risk for the development of SSc in patients with RP. To access this question prospective long-term studies in high numbers of patients including an analysis of ocular microvasculature are needed.

Another interesting finding of our study is the significant inverse correlation between the skin score and the VD data of the SCP. Recently, a correlation between oral vascular density and skin scores using a novel non-invasive imaging tool has been described, supporting both that microvascular changes are a general and pathogenetic relevant phenomenon in SSc and the usefulness of novel quantitative imaging microvascular techniques^7^.

Our study has some limitations: First, it is a cross-sectional study. Therefore, we cannot comment on the value of VD measurements for evaluation of disease progression or changes of VD values during vasoactive treatment. Further studies on OCTA imaging with a longitudinal design should be performed in future. A second limitation is the small sample size, especially of the VEDOSS group. Nevertheless, considering the rarity of the disease, our OCTA study is the one with the largest number of SSc patients included and the patients in the current study accounted a rather homogeneous study population. Moreover, in our study we focused on retinal and choriocapillary microcirculation using the Optovue XR Avanti OCTA-device. Using this device EDI-OCT imaging is not possible, therefore we cannot reliably comment on choroidal thickness and substructures of the different choroidal layers. However, Ranjbar et al. have described structural changes of the choroid and its layers as the consequences of the microcirculation damages using EDI-OCT imaging in SSc patients with dcSSc and lcSSc^10^. Rommel et al. showed a significantly lower macular volume in SSc patients as well as a correlation between disease duration and retinal and choroidal malperfusion, indicating an involvement of both tissues in late stages of the disease^31^.

In conclusion, patients with SSc show an altered ocular perfusion. Results of our study demonstrated a reduced VD in the macula, choriocapillaris and optic nerve head in SSc patients compared to healthy controls. In patients with a very early disease (VEDOSS), choriocapillary perfusion was already significantly reduced. Moreover, the OCTA data also correlated with the disease severity evaluated using nailfold capillaroscopy and the mRSS. Further studies with a larger number of patients are needed to underline the clinical utility of this technology in identifying patients at high risk for developing systemic involvement and to elaborate the diagnostic and prognostic value of these microvascular changes.

## Supplementary Information

Supplementary Table 1.

Supplementary Table 2.

Supplementary Table 3.
