# Supplementary material for: Altered ocular microvasculature in patients with systemic sclerosis and very early disease of systemic sclerosis using optical coherence tomography angiography
Source: Sci Rep. 2022 Jun 29;12:10990. doi: 10.1038/s41598-022-14377-6 (PMC9243093; doi:10.1038/s41598-022-14377-6)
Supplement: Supplementary file 1 — Supplementary Table 1. [file 41598_2022_14377_MOESM1_ESM.docx]

**Supp. Table 1** Detailed data of the patient group including laboratory data, clinical data, ocular symptoms and history of cardiovascular diseases

|  | **Patient** | **SSc type** | **Laboratory data** | | | | | | | | **Ocular symptoms** | | | |
| --- | --- | --- | --- | --- | --- | --- | --- | --- | --- | --- | --- | --- | --- | --- |
|  |  |  | **specific antibodies (FIA)** | **ANA (IFT)** | **fluorescence  pattern** | **other** | **RF (IU/ml)** | **anti-CCP**  **(U/ml)** | **CRP (mg/dl)** | **WBC in 10³/mcL** | KC sicca  (LIPCOF; MGD) | madarosis | eyelid  stiffness | eyelid tele- angiectasia |
| **SSc** | 1 | diffuse | scl70 >240 U/ml | ANA >1:5120 | fine speckled,  chromosome + | anti-Ro/SSA | nd | nd | 1.1 | 8 | yes | yes | yes | yes |
|  | 2 | diffuse | scl70, titer nd | ANA 1:2560 | nd | dsDNA, ATR1, ETAR | nd | nd | <0.5 | 6 | yes | no | no | no |
|  | 3 | diffuse | scl70 >240 U/ml | ANA 1:2560 | fine speckled,  chromosome + |  | nd | nd | nd | 4.8 | yes | no | no | no |
|  | 4 | diffuse | scl70 >240 U/ml | ANA 1:5120 | fine speckled,  chromosome + |  | 10.4 | nd | <0.5 | 7 | no | no | no | no |
|  | 5 | limited | scl70 160 U/ml | ANA 1:320 | nd |  | nd | nd | 0.9 | 6 | yes | no | no | no |
|  | 6 | limited | scl70 >240 U/ml | ANA 1:5120 | fine speckled,  chromosome - | anti-Ro/SS-A | <9.9 | 0,6 | 1 | 9 | yes | yes | yes | yes |
|  | 7 | diffuse | no specific ab | ANA 1:5120 | fine speckled,  chromosome - |  | nd | nd | 2 | 6 | yes | yes | yes | yes |
|  | 8 | diffuse | scl70 >240 U/ml | ANA 1:5120 | homogeneous,  chromosome + |  | <9.9 | 0,6 | <0.5 | 8 | no | no | no | no |
|  | 9 | limited | no specific ab | ANA positive,  titer nd | fine speckled,  chromosome - |  | nd | nd | <0.5 | 6 | no | no | no | no |
|  | 10 | limited | CENP-B 240.00 U/ml | ANA positive,  titer nd | centromere,  chromosome + |  | 9.8 | nd | 0.6 | 4.5 | no | no | no | no |
|  | 11 | diffuse | scl70, titer nd | ANA 1:2560 | homogeneous | cryoglobulines | nd | nd | 1.3 | 10 | yes | no | yes | yes |
|  | 12 | limited | CENP-B 93.00 U/ml | ANA 1:5120 | centromere,  chromosome + |  | 9.5 | nd | <0.5 | 6 | yes | no | yes | no |
|  | 13 | limited | anti-fibrillarin-ab 175.00 U/ml | ANA >1:5120 | nucleolar,  chromosome - | PL-7-AK (borderline) | 10.4 | nd | 0.6 | 8 | no | no | no | no |
|  | 14 | limited | CENP-B >240 U/ml | ANA 1:5120 | centromere,  chromosome + | anti-CENP-A,  anti-Ro 52 | nd | nd | 0.7 | 9 | yes | no | no | no |
|  | 15 | diffuse | scl70 >240 U/ml | ANA 1:5120 | fine speckled,  chromosome + | anti-Ku,  anti-RO/SS-A | <7 | <5 | <0.5 | 5.5 | yes | no | no | no |
| **VEDOSS** | 16 | n/a | scl70, titer nd | ANA 1:3200 | granular, mitosis - | SS-A, SS-B,  anti-Ro 52 | 20.1 | nd | <0.5 | 5.7 | yes | no | no | no |
|  | 17 | n/a | CENP-B 147.00 U/ml | ANA >1:5120 | centromere,  chromosome + |  | <9.9 | 0.4 | <0.5 | 7 | no | no | no | no |
|  | 18 | n/a | CENP-B 240.00 U/ml | ANA 1:5120 | centromere,  chromosome + |  | <9.9 | 0.5 | <0.5 | 5 | yes | no | no | no |
|  | 19 | n/a | CENP-B 177.00 U/ml | ANA 1:5120 | centromere,  chromosome + |  | nd | nd | <0.5 | 4.3 | yes | no | no | no |
|  | 20 | n/a | CENP-B >240.00 U/ml | ANA >1:5120 | centromere,  chromosome + |  | <20 | 0.2 | <0.5 | 5.1 | no | no | no | no |
|  | 21 | n/a | no specific ab | ANA 1:320 | nucleolar,  chromosome - |  | negative  (<30) | nd | <0.5 | 6 | no | no | no | no |
|  | 22 | n/a | no specific ab | ANA 1:160 | fine speckled,  chromosome - | cold agglutinins 2008 (not detectable 2015) | 10 | 0.9 | <0.5 | 6 | yes | no | no | no |

*SSc,* systemic sclerosis; *VEDOSS,* very early disease of systemic sclerosis; *ab,* antibodies; *FIA*, fluorescence immunoassay; *ANA,* antinuclear antibodies; *KC,* keratoconjunctivitis; *LIPCOF*, lid-parallel conjunctival folds; *MGD*, meibomian gland dysfunction; *scl70*, scl-70-antibody; *PAH*, pulmonary arterial hypertension; *AV-B*, atrioventricular block; *WBC,* white blood cell count; *IFT*, immune fluorescence test; *nd,* not determined; *mRss,* modified Rodnan skin score; *PI*, pulmonary involvement (CT-graphic); *DLCO,* Diffusion Capacity of the Lungs for Carbon Monoxide; *CHF*, chronic heart failure; *PVD,* peripheral vascular disease; *FVC,* forced vital capacity, *GC*, giant capillaries;

**Supp. Table 2** (continued)

|  | **Patient** | **SSc type** | **Clinical data** | | | | | | | | | | **Cardiovascular diseases** | | |
| --- | --- | --- | --- | --- | --- | --- | --- | --- | --- | --- | --- | --- | --- | --- | --- |
|  |  |  | **mRss** | **skin involvement** | **nailfold capillaroscopy** | **Raynaud** | **ulcer (hand, feet)** | **PI** | **DLCO  single Breath** | **FVC (%)** | **Involvement of other organs** | **joint  problems** | **arterial  hypertension** | **nicotine  abuse** | **cardiac diseases** |
| **SSc** | 1 | diffuse | 41 | teleangiectasia | nd | yes | yes | fibrosis | 58.8% | 55 | PAH, oesophagus,  renal | yes | no | no | first-degree  AV-B |
|  | 2 | diffuse | 4 | teleangiectasia | ectasia | yes | yes | fibrosis | 62.2% | 80 | oesophagus | no | yes | no | paroxysmal  tachycardia |
|  | 3 | diffuse | 2 | no | ectasia, haemorrhages | yes | no | fibrosis | 81.5% | 59 | no | yes | yes | no |  |
|  | 4 | diffuse | 26 | teleangiectasia | haemorrhages, ramifications | yes | yes | fibrosis | not possible | 36 | oesophagus | yes | no | no |  |
|  | 5 | limited | 7 | no | ectasia, haemorrhages | yes | no | fibrosis | 102.5% | 74 | no | yes | yes | no |  |
|  | 6 | limited | 6 | calcinosis | nd | yes | no | fibrosis | 61.1% | 90 | oesophagus | yes | yes | yes |  |
|  | 7 | diffuse | 27 | teleangiectasia | GC | yes | yes | fibrosis | 34.0% | 77 | oesophagus | yes | no | yes |  |
|  | 8 | diffuse | 11 | no | nd | yes | yes | fibrosis | 89.0% | 55 | no | no | no | no |  |
|  | 9 | limited | 11 | teleangiectasia, calcinosis | GC | yes | yes | no | 56.6% | 88 | oesophagus | no | no | yes | CHF  PVD |
|  | 10 | limited | 2 | no | nd | yes | yes | fibrosis | 58.7% | 94 | oesophagus | yes | no | no |  |
|  | 11 | diffuse | 39 | teleangiectasia | nd | yes | yes | fibrosis | 47.1% | 101 | no | no | yes | yes | PVD |
|  | 12 | limited | 6 | teleangiectasia | nd | yes | no | no | 82.9% | 87 | no | yes | no | no |  |
|  | 13 | limited | 15 | teleangiectasia, calcinosis | GC, haemorrhages, ectasia, multiple ramifications, | yes | yes | no | 61.0% | 72 | PAH, oesophagus | yes | yes | no |  |
|  | 14 | limited | 2 | teleangiectasia | GC, ramifications | yes | no | fibrosis | 45.9% | 82 | PAH | no | yes | no |  |
|  | 15 | diffuse | 2 | teleangiectasia | GC, ramifications | yes | yes | alveolitis/  dystelectasis | 94.4% | 103 | no | yes | no | no |  |
| **VEDOSS** | 16 | n/a | 2 | no | ectasia, multiple ramifications | yes | no | suspected | 87.6% | 94 | no | unknown | no | no |  |
|  | 17 | n/a | 0 | no | nd | yes | no | no | 79.2% | 96 | no | yes | no | no |  |
|  | 18 | n/a | 1 | teleangiectasia | nd | yes | no | no | 80.8% | 88 | no | yes | yes | no |  |
|  | 19 | n/a | 0 | no | GC, ectasia, haemorrhages | yes | no | no | 82.9% | 68 | no | yes | no | no |  |
|  | 20 | n/a | 0 | no | nd | yes | no | no | 80.4% | 75 | no | no | no | no |  |
|  | 21 | n/a | 2 | no | GC, haemorrhages, ramifications, ectasia | yes | no | no | 93.3% | 111 | no | no | no | no |  |
|  | 22 | n/a | 0 | no | GC, haemorrhages, ectasia, multiple ramifications | yes | no | no | 101.5% | 93 | no | unknown | no | no |  |

*SSc,* systemic sclerosis; *VEDOSS,* very early disease of systemic sclerosis; *ab,* antibodies; *FIA*, fluorescence immunoassay; *ANA,* antinuclear antibodies; *KC,* keratoconjunctivitis; *LIPCOF*, lid-parallel conjunctival folds; *MGD*, meibomian gland dysfunction; *scl70*, scl-70-antibody; *PAH*, pulmonary arterial hypertension; *AV-B*, atrioventricular block; *WBC,* white blood cell count; *IFT*, immune fluorescence test; *nd,* not determined; *mRss,* modified Rodnan skin score; *PI*, pulmonary involvement (CT-graphic); *DLCO,* Diffusion Capacity of the Lungs for Carbon Monoxide; *CHF*, chronic heart failure; *PVD,* peripheral vascular disease; *FVC,* forced vital capacity, *GC*, giant capillaries;
