# Supplementary material for: Altered ocular microvasculature in patients with systemic sclerosis and very early disease of systemic sclerosis using optical coherence tomography angiography
Source: Sci Rep. 2022 Jun 29;12:10990. doi: 10.1038/s41598-022-14377-6 (PMC9243093; doi:10.1038/s41598-022-14377-6)
Supplement: Supplementary file 2 — Supplementary Table 2. [file 41598_2022_14377_MOESM2_ESM.docx]

**Supp. Table 2.** Correlation analysis between OCTA parameters (vessel density, %) and clinical parameters (modified Rodnan skin score, *mRss* and nailfold capillary density, *NCD*). Skin score was obtained in the patient group only (n = 22), nailfold capillary density was obtained in both patient and control group (n = 44). *rSp = Spearman correlation coefficient; SCP = superficial capillary plexus; DCP = deep capillary plexus; RPC = radial peripapillary capillary; CC = choriocapillaris*

| **OCTA parameter** | **Clinical parameter** | **rSp** | **p-value** |
| --- | --- | --- | --- |
|  |  |  |  |
| SCP whole en face | mRss | -0.504 | **0.017** |
| SCP fovea | mRss | -0.068 | 0.765 |
| SCP parafovea | mRss | -0.433 | **0.044** |
| DCP whole en face | mRss | -0.133 | 0.557 |
| DCP fovea | mRss | -0.053 | 0.814 |
| DCP parafovea | mRss | -0.155 | 0.492 |
| RCP whole en face | mRss | -0.350 | 0.110 |
| RCP inside disc | mRss | -0.002 | 0.994 |
| RCP peripapillary | mRss | -0.356 | 0.104 |
| CC | mRss | -0.031 | 0.891 |
|  |  |  |  |
| SCP whole en face | NCD | 0.342 | 0.064 |
| SCP fovea | NCD | 0.398 | **0.030** |
| SCP parafovea | NCD | 0.255 | 0.174 |
| DCP whole en face | NCD | 0.143 | 0.450 |
| DCP fovea | NCD | 0.148 | 0.437 |
| DCP parafovea | NCD | 0.174 | 0.357 |
| RCP whole en face | NCD | 0.235 | 0.211 |
| RCP inside disc | NCD | 0.278 | 0.137 |
| RCP peripapillary | NCD | 0.163 | 0.391 |
| CC | NCD | 0.456 | **0.011** |
