# Supplementary material for: Altered ocular microvasculature in patients with systemic sclerosis and very early disease of systemic sclerosis using optical coherence tomography angiography
Source: Sci Rep. 2022 Jun 29;12:10990. doi: 10.1038/s41598-022-14377-6 (PMC9243093; doi:10.1038/s41598-022-14377-6)
Supplement: Supplementary file 3 — Supplementary Table 3. [file 41598_2022_14377_MOESM3_ESM.docx]

**Supplemental Tables 3a and 3b.** Vessel density (VD, %) of the SSc subgroups. (a: definite SSc, b: VEDOSS) compared to the control group obtained in the regions indicated (superficial retinal capillary plexus (SCP), choriocapillaris (CC) and optic nerve head capillary density (RPC)), as well as the foveal avascular zone area (FAZ). Data are presented as median [25^th^, 75^th^ percentile]. A Mann-Whitney-U-test was used to compare the groups. Bold: statistically significant differences.

**Supp. Table 3a.**

| OCTA |  | VD control group | VD definite SSc | p-value |
| --- | --- | --- | --- | --- |
| SCP | whole en face | 45.25 [43.90, 48.23] | 43.10 [39.95, 46.90] | **0.015** |
|  | fovea | 18.55 [15.58, 22.08] | 17.25 [11.40, 18.73] | 0.092 |
|  | parafovea | 47.60 [46.15, 50.25] | 46.10 [42.15, 49.65] | **0.030** |
| CC |  | 116.96 [114.19, 118.74] | 111.07 [104.39, 114.61] | **0.003** |
| RPC | whole en face | 50.75 [48.48, 51.55] | 49.90 [47.63, 51,55] | 0.112 |
|  | inside disc | 52.80 [49.68, 55.05] | 47.05 [42.08, 53.35] | **0.026** |
|  | peripapillary | 53.00 [50.88, 54.60] | 53.10 [50.75, 54.70] | 0.383 |
| FAZ |  | 0.24 [ 0.17, 0.31] | 0.26 [0.18, 0.33] | 0.350 |

**Supp. Table 3b**.

| OCTA |  | VD control group | VD VEDOSS | p-value |
| --- | --- | --- | --- | --- |
| SCP | whole en face | 45.25 [43.90, 48.23] | 44.80 [42.80, 37.20] | 0.566 |
|  | fovea | 18.55 [15.58, 22.08] | 17.00 [10.80, 22.20] | 0.328 |
|  | parafovea | 47.60 [46.15, 50.25] | 47.90 [45.90, 50.30] | 0.901 |
| CC |  | 116.96 [114.19, 118.74] | 107.14 [103.77, 113.42] | **0.008** |
| RPC | whole en face | 50.75 [48.48, 51.55] | 51.10 [50.50, 53.00] | 0.217 |
|  | inside disc | 52.80 [49.68, 55.05] | 47.40 [46.20, 53.30] | 0.181 |
|  | peripapillary | 53.00 [50.88, 54.60] | 54.10 [52.90, 56.70] | 0.135 |
| FAZ |  | 0.24 [ 0.17, 0.31] | 0.23 [0.18, 0.32] | 1,000 |
